# Mental Health among African American and Latinx Men who have sex with men after the COVID-19 Lockdown in Los Angeles – Findings from the HOPE cohort

**DOI:** 10.1007/s10597-022-00970-6

**Published:** 2022-04-20

**Authors:** Yan Wang, Janni Kinsler, William G. Cumberland, Sean D. Young

**Affiliations:** 1 Division of Infectious Diseases, David Geffen School of Medicine, University of California, Los Angeles (UCLA), 10833 Le Conte, 90095 Los Angeles, California USA; 2 Section of Pediatric Dentistry, School of Dentistry, University of California, Los Angeles (UCLA), 10833 Le Conte, 90095 Los Angeles, California USA; 3 Department of Biostatistics, Fielding School of Public Health, University of California, Los Angeles (UCLA), 10833 Le Conte, 90095 Los Angeles, California USA; 4 Department of Emergency Medicine, University of California, 92697 Irvine, CA USA; 5 Department of Informatics, Institute for Prediction Technology, University of California, 6091 Bren Hall, Irvine, CA USA

**Keywords:** COVID-19, Mental health, MSM, Food security, Anxiety, Depression

## Abstract

This study aimed to examine the depression and anxiety among men of color (primarily African American and Latinx) who have sex with men after the lockdown due to the COVID-19 pandemic. Outcomes included 21-item Beck Depression Inventory (BDI), 7-item Generalized Anxiety Disorder (GAD), and a 10-item COVID-related anxiety measure using a modified H1N1-related anxiety question. Independent variables were food insecurity and belief in government efficiency. Data were analyzed by Regression models with random cluster effects. Food insecurity experiences were significantly associated with higher depression (p < 0.001), higher anxiety (p < 0.001), and higher pandemic-related anxiety (p < 0.001). Higher levels of belief in government efficiency were significantly associated with lower depression (p < 0.05), less anxiety (p < 0.05), and less pandemic-related anxiety (p-value < 0.001). These findings emphasize the importance of establishing trust between government and at-risk communities when issuing public health policies, especially during unforeseen circumstances, as well as to ensure basic human rights, such as food security.

## Introduction

The HIV infection rate among men who have sex with men (MSM) continues to be disproportionately high (Crepaz et al., 2019). In the United States, MSM are the population most affected by HIV. In 2019, MSM made up 69% of new HIV diagnoses in the US. Among all MSM, African American MSM accounted for more than 37.9% of new HIV diagnoses and Hispanic/Latino MSM accounted for 32.5% of new HIV diagnoses (Control & Prevention, 2021). MSM of color (cMSM) receive inadequate HIV-related preventive care and social and mental health support services (Crepaz et al., 2019). Studies have shown that MSM/cMSM populations in the US suffer from high rates of psychosocial issues, such as depression, anxiety, substance abuse and sexual abuse (Batchelder et al., 2017; Jie et al., 2012) which are associated with a high incidence of HIV infection, transmission, and lower levels of health care engagement (Batchelder et al., 2017). Prior to the COVID-19 pandemic, resources were lacking to address the psychological and social barriers among cMSM. With the onset of the COVID-19 pandemic, additional challenges to addressing psychological and social issues among the MSM population arose due to the COVID-19 pandemic mandates, including the lockdown, social distancing for an indefinite period, stay-at-home orders and quarantine policies (Pfefferbaum & North, 2020). For example, long periods of social isolation and loneliness during the pandemic were found to be associated with people’s health status. Studies showed that social isolation significantly increased the risk of death due to the potential increase of smoking, obesity and physical inactivity (National Academies of Sciences & Medicine, 2020); and loneliness was associated with high rates depression, anxiety, and even suicide (Courtet et al., 2020; Sher, 2020).

While studies have recently shown the impact of anxiety and depression as a result of COVID-19 in the general population (Hyndman et al., 2021; Millar et al., 2020; Torres et al., 2020; Xiong et al., 2020), there is little research on anxiety and depression as a result of COVID-19 among MSM/cMSM (Singh et al., 2020; Wheaton et al., 2012). There are also no available psychometric validated instruments that can be used to measure pandemic-related anxiety and depression. All current studies in the literature have used existing scales to measure mental health status (Camargo et al., 2021; Pan et al., 2021; Sanchez et al., 2020; Septarini et al., 2021). For example, Sanchez et al. conducted a rapid online survey in April of 2020 among 1,051 MSMs in the United States (Sanchez et al., 2020). They found that COVID had a negative impact on general well-being, social interactions, money, food, alcohol and substance use. The survey focused on the interruption of services and sexual behaviors, such as HIV testing and treatment, or condom use. The majority of the survey participants were non-Hispanic White (70%). Thus, there is still a need to study the effect the COVID-19 pandemic is having on the mental health of cMSM.

The purpose of this study was to examine factors affecting mental health among a cohort of cMSM in Southern California during the COVID pandemic lockdown issued on March 19, 2020. The cohort of cMSM is part of an ongoing longitudinal clustered randomized clinical trial called Harnessing Online Peer Education (HOPE) (Young et al., 2014). The HOPE study aims to evaluate whether a social media peer-led HIV prevention intervention could be used to increase HIV-testing among African American and Latino MSM. In this paper, we tested the following hypotheses: high level of food security and belief in government efficiency are associated with lower levels of depression and anxiety.

## Methods

### Participants

Participants for this study included 300 cMSM who were recruited from the ongoing HOPE cohort between July 8, 2020 and July 14, 2020. All HOPE participants were 18 years of age or older with HIV negative or serostatus unknown at the time of enrollment. They all were MSM (primarily cMSM) who were randomly assigned to a control condition or intervention condition with peer-delivered HIV information in private Facebook groups given to the intervention subjects. Participants were followed at 3 months, 6 months and 12 months post intervention. For this study, we sent the recruitment link to all 840 participants who were in the HOPE cohort for at least 12 weeks, and recruited the first 300 participants who responded by selecting the box stating their agreement to participate in the study. The study was approved by the UCLA IRB.

### Data collection

The survey was conducted using SurveyMonkey Inc (*SurveyMonkey Inc.*, 2020) during the first peak of the COVID-19 pandemic. Survey questions were focused on participants’ experiences, behavior change, and mental health status during the pandemic. Most behavior-related questions were assessed in the time frame of the past three months, while mental health-related questions were assessed for the past two weeks. In this way, we were able to estimate the association between the survey responses and mental health status after the lockdown.

### Mental Health Outcomes

The primary outcomes were anxiety and depression. We used two instruments to measure anxiety. In the first instrument, we assessed the level of anxiety as a result of COVID-19. The instrument was a modified 10-item questionnaire derived from similar items used to measure anxiety to the H1N1 influenza in 2009–2010 (Wheaton et al., 2012) and the Zika virus pandemic during 2015–2016 (Blakey & Abramowitz, 2017). Subjects’ agreement to each statement was on a 5-point Likert scale ranging from 0 (‘‘very low’’ or “not at all”) to 4 (‘‘very high’’ or “extremely”). A higher score represented higher anxiety. Table 1 presented these ten questions.

**Table 1:** Item properties of COVID-19 related anxiety

Items of SARS-CoV2 related anxiety (higher score indicated higher anxiety level)	Mean (SD) Range 0–4	Standardized Alpha with deleted variable	Correlation with Total
1. To what extent are you concerned about Coronavirus?	2.82 (1.08)	0.78	0.64
2. To what extent do you believe that Coronavirus could become a “pandemic” in the U.S.?	3.73 (0.65)	0.78	0.57
3. How likely is it that you could become infected with Coronavirus?	2.24 (0.99)	0.81	0.34
4. How likely is it that someone you know could become infected with Coronavirus?	2.82 (0.95)	0.80	0.44
5. How quickly do you believe contamination from Coronavirus is spreading in the U.S.?	3.38 (0.92)	0.78	0.59
6. How knowledgeable do you feel about Coronavirus?	2.65 (0.9)	0.82	0.22
7. If you did become infected with Coronavirus, to what extent are you concerned that you will be severely ill?	2.51 (1.18)	0.80	0.46
8. To what extent has the threat of Coronavirus influenced your decisions to be around people?	3.22 (1)	0.78	0.62
9. To what extent has the threat of Coronavirus influenced your travel plans?	3.52 (0.94)	0.80	0.46
10. To what extent has the threat of Coronavirus influenced your use of safety behaviors (e.g., hand sanitizer)?	3.52 (0.72)	0.79	0.55
Total Score (with range 0–40)	**30.4 (5.7)**	Overall alpha = 0.81	
Correlation with score of Generalized Anxiety Disorder 7-item	0.38***		
Correlation with score of Beck Depression Inventory	0.23***		

* <0.05, ** <0.01, *** <0.0001

We evaluated internal consistency using standardized Cronbach’s alpha statistic and correlation with total score. The overall standardized Cronbach’s coefficient alpha greater than 0.7 suggested high internal validity (Bland & Altman, 1997). We reported the standardized alpha coefficient with deleted variables to measure how each variable reflected the consistency of the scale. If the standardized alpha decreased after removing a variable from the construct, then this variable was found to be strongly correlated with other variables in the scale. On the other hand, if the standardized alpha increased after removing a variable from the construct, then removing this variable from the scale made the construct more reliable (Cronbach, 1951). A higher value of an item’s correlation with the total (> 0.3) indicated the item correlated very well with the overall scale (Bernstein, 2010).

The overall standardized Cronbach’s coefficient alpha of COVID-19 related anxiety was 0.81 (Table 1). The standardized alpha coefficients with deleted variables ranged from 0.78 to 0.82. Removing item 6, “How much exposure have you had to information about Coronavirus?”, increased the overall standardized Cronbach’s coefficient alpha to 0.82. Also, only item 6 had a low correlation (0.22) with the total, and hence was excluded when calculating the score of COVID-19 related anxiety. A higher total score indicated higher anxiety related to COVID-19.

The second instrument used to measure anxiety was the Generalized Anxiety Disorder 7-item (GAD-7) scale (Spitzer et al., 2006), which contained 7 items whose total ranged from 0 to 21. A threshold of 10 for GAD-7 was used to screen for anxiety disorder. Depression was measured using the Beck Depression Inventory (BDI), which included 21 items with a 4-point scale. The summation of all scores ranged from 0 to 63 with higher score indicating higher level of depression. In non-clinical populations, depression is defined as a score 20 and above (Jackson-Koku, 2016).

## Other variables

We collected demographics, such as age, race/ethnicity of the cMSM, education, and marital status. We surveyed the participants’ income in the past month, current employment status, whether they were essential workers (essential workers included those working in public health or health care, law enforcement, public safety, first responders, food and agriculture, energy and electricity, petroleum, water and waste, transportation, public works, communications), and whether they were able to work remotely during the lockdown. Alcohol use was summarized from the following three variables: (1) on average, how many whole alcoholic drinks do you have each week; (2) if you are male, how many days have you had five or more drinks in one day in the past three months; and (3) if you are female, how many days have you had four or more drinks in one day in the past three months. We also measured whether they used alcohol more than four drinks on a typical day, and whether they used tobacco, marijuana, or any illicit drugs in the past three months. Food insecurity was a dichotomized variable equal to 1 if a respondent answered yes to any of the following six questions: (1) had difficulty in getting needed groceries, (2) was afraid to go to the store, (3) asked others to get groceries, (4) was worried about running out of food, (5) had no money to get enough food, and (6) could not afford to eat balanced meals. We collected general health status, whether the participant visited a provider, whether participant used telemedicine or telehealth, and difficulties in prescription (PrEP or other medications) in the past three months.

The beliefs in government efficiency were measured using eight variables related to both the HIV pandemic and COVID-19 pandemic (Table 2). The overall Cronbach’s alpha coefficient was 0.85 (> 0.7 criteria for internal consistency). The eight items on belief in government efficiency on handling the HIV epidemic and COVID-19 pandemic had the alpha coefficients with deleted variables ranged from 0.80 to 0.91. All eight items were significantly associated with pandemic-related anxiety with correlation total except for the item, “How compliant have you been in following government guidelines”. The overall Cronbach’s alpha coefficient after removing this question increased to 0.91. This item was excluded when calculating the total belief score.

**Table 2:** Item properties of belief in government efficiency on public health crises

Belief in government efficiency	Mean (SD) Range 0–4	Standardized Alpha with deleted variable	Correlation with Total
How well of a job do you feel your government has done in addressing HIV?	1.25 (1.12)	0.82	0.67
How trustful are you that your government will effectively address HIV?	1.14 (1.07)	0.81	0.72
How well do you feel your government is equipped with the necessary resources to address HIV?	1.6 (1.17)	0.82	0.64
How well of a job do you feel your government has done in addressing COVID-19?	0.73 (1.07)	0.81	0.72
How trustful are you that your government will effectively address COVID-19?	0.81 (1.01)	0.8	0.8
How well do you feel your government is equipped with the necessary resources to address COVID-19?	1.09 (1.2)	0.81	0.71
How compliant have you been in following government guidelines? ^#^	3.06 (1.02)	0.91	-0.17
How confident do you feel your government would be able to address future public health crisis?	1.1 (1.09)	0.81	0.71
Total score	7.72 (6.23)	Overall alpha = 0.85	

* <0.05, ** <0.01, *** <0.0001

^#^ Item was excluded when calculating the total score due to correlation with total < 0.3

### Statistical analysis

We compared all the demographic variables among the 300 participants and the rest of the HOPE cohort at baseline to determine whether the 300 participants were different from the rest of cohort. If any variable was significantly different between these two groups at baseline, we included that variable in the regression analysis. We reported the characteristics of these 300 participants. We applied regression models with a random cluster effects to allow for possible correlations among those participants in the same Facebook group. In the model, we adjusted for demographic characteristics, including age, race/ethnicity, education, marital status, income level, employment status, general health, food insecurity and belief in government efficiency. We reported regression coefficients and their 95% confidence intervals. All statistical analysis was conducted using SAS 9.4.

## Results

### Characteristics of the sample

The mean age of participants was 33.7 with a range of 21 to 65 as shown in Table 3. Most participants had a college degree (77%). The majority of participants (70%) were single. Over half (57%) of the participants worked more than 35 h per week in the past month. Approximately one third of participants (38%) were essential workers and almost half (49%) worked remotely. Only 20% of participants had an income that was less than $1,000 in the past month. Over three quarters of participants (77%) experienced food insecurity in the past three months and nearly half (43%) of participants reported having more than 4 drinks on one typical day.

**Table 3:** Characteristics of the participants

Variables	Statistics (N = 300), mean(SD) or N(%)
Age	33.7 (8.9), range 21–65
Intervention	
Control	145 (48.3%)
Intervention	155 (51.7%)
Complete study	
Yes	192 (64%)
Still in cohort	108 (36%)
Race/Ethnicity	
White/European Descent	0 (%)
Black/African American	54 (18%)
American Indian or Alaska Native	11 (3.7%)
Asian or Pacific Islander	18 (6%)
Latino/Caribbean	188 (62.7%)
Other	29 (9.7%)
Education	
High school or less	70 (23.3%)
Associate or bachelor’s degree	154 (51.3%)
Graduate and above	76 (25.3%)
Marital status	
Single (never married)	209 (69.7%)
Married or domestic partnership	91 (30.3%)
Legal income in the past month	
$1000 or less	57 (19%)
>$1000-$2000	60 (20%)
>$2000-$3000	58 (19.3%)
>$3000-$4000	46 (15.3%)
>$4000-$5000	24 (8%)
$5000 or more	42 (14%)
Current work	
Full time (35 + hours/week)	171 (57%)
Part time (< 35 h/week)	41 (13.7%)
Not working	88 (29.3%)
Essential worker	
Yes	113 (37.7%)
No	99 (33%)
Being able to work remotely	
Yes	147 (49%)
No	65 (21.7%)
Tobacco (past 3 months)	
Yes	75 (25%)
No	225 (75%)
Alcohol (4 + drinks on a typical day)	
Yes	141 (47%)
No	159 (53%)
Substance use	
Not use any	136 (45.3%)
Marijuana	149 (49.7%)
Other	15 (5%)
Food insecurity	
Yes	232 (77.3%)
No	68 (22.7%)

Table 4 presents the self-reported health-related status of participants. About half of the participants (47.7%) rated their general health as healthy in the survey. Approximately one quarter (23%) of cMSM in our study reported depression, with BDI scale mean of 13.1 (std 10.6) and 30.7% MSM reported anxiety, with GAD-7 scale mean of 8.3 (std 5.9). More than half (56.7%) of the participants had visited their provider in the past three months. Among them, about a third (32.3%) used telehealth for their physical health and 13.3% used telehealth for their mental health in the past three months. The COVID-19 positive rate (as reported by the provider) was 3.7% among the participants.

**Table 4:** Health-related statistics status in the past three months

Variables	Statistics (N = 300), N(%)
General Health	
Healthy	143 (47.7%)
Unhealthy	45 (15%)
Average	111 (37%)
Don’t know	1 (0.3%)
Depression (Beck Depression Inventory > 20)	
Yes	69 (23%)
No	231 (77%)
Anxiety (Generalized Anxiety Disorder > 7)	
Yes	92 (30.7%)
No	208 (69.3%)
Visit health provider in the past 3 months	
Yes	170 (56.7%)
No	130 (43.3%)
Telemedicine/telehealth in the past 3 months	
No	186 (62%)
Yes - physical health	97 (32.3%)
Yes - mental health	40 (13.3%)
COVID-19 (told by provider)	
Yes	11 (3.7%)
No	289 (96.3%)

Table 5 presents the results of the regression models with ranom group effects. In this table, each column was a model for the mental health-related outcomes, depression, anxiety, and COVID-related anxiety. Food insecurity experiences were significantly associated with higher depression (p < 0.001), higher anxiety (p < 0.001), and higher pandemic-related anxiety (p < 0.001). Especially, experiencing food insecurity during the lockdown was associated with 5.16 higher BDI depression level (P < 0.001), 4.0 higher GAD anxiety level (p < 0.001), 3.2 score higher of COVID-19 anxiety compared to those with food security (p < 0.001). The higher level of belief in government on handling the pandemic was significantly associated with a lower depression score (p = 0.047), less anxiety (p = 0.019), and less pandemic-related anxiety (p < 0.001). Those who followed COVID-related governmental guidelines to the extreme had higher pandemic-related anxiety (< 0.001) when compared to those who followed the guidelines moderately. Those who used tobacco had higher levels of depression (p < 0.001). Those who rated themselves as unhealthy had higher levels of BDI depression (p < 0.001) and higher GAD anxiety (p < 0.001). In the appendix table S1, we used categorical depression level and anxiety level using cut-off scores for general population.

**Table 5:** Regression of depression (Beck Depression Inventory), anxiety (Generalized Anxiety Disorder) and pandemic anxiety (COVID-10, 10 items of COVID-19 related anxiety)

	BDI-21	GAD-7	COVID-10
Intervention			
Yes	Reference		
No	-0.26 (-2.41,1.89)	0.77 (-0.44,1.98)	0.99 (-0.12,2.11)
Age			
One year increase	-0.08 (-0.18,0.02)	-0.03 (-0.09,0.03)	0.05 (0,0.11)
Education			
High School or less	0.66 (-2.5,3.82)	0.43 (-1.35,2.21)	0.41 (-1.23,2.04)
Associate/Bachelor degree	2.23 (-0.41,4.87)	1.46 (-0.03,2.94)	-0.25 (-1.62,1.12)
Graduate and above	Reference		
Marital status			
Married/in a partnership	-1.38 (-4.01,1.25)	-0.23 (-1.72,1.25)	1.27 (-0.1,2.63)
Single	Reference		
Employment			
Other	3.46 (0.93,5.99)**	0.78 (-0.64,2.21)	-0.64 (-1.95,0.67)
Part time	1.83 (-1.43,5.09)	0.16 (-1.68,1.99)	-1.42 (-3.1,0.27)
Full time	Reference		
General health			
Unhealthy	5.92 (2.68,9.17)***	3.11 (1.29,4.94)***	1.61 (-0.08,3.29)
Average	2.25 (-0.08,4.57)	0.87 (-0.44,2.18)	1.32 (0.12,2.53)*
Healthy	Reference		
Use Tobacco			
Yes	4.57 (2.09,7.05)***	1.26 (-0.13,2.66)	-0.49 (-1.78,0.79)
No	Reference		
Ever experienced food insecurity			
Yes	5.16 (2.5,7.82)***	3.99 (2.49,5.48)***	3.24 (1.86,4.62)***
No	Reference		
Hours on social media/online communities			
4–8 h	1.51 (-0.84,3.86)	0.03 (-1.3,1.35)	0.18 (-1.03,1.4)
More than 8 h	4.75 (1.17,8.33)**	2.39 (0.38,4.41)*	1.72 (-0.14,3.57)
<4 h	Reference		
Belief in government efficiency			
7-item score	-0.18 (-0.36,0)*	-0.12 (-0.22,0.02)*	-0.23 (-0.32,0.13)***
Follow government guidelines			
Not at all	1.5 (-2.66,5.65)	0.03 (-2.31,2.37)	-1.77 (-3.92,0.38)
Moderately	0.86 (-2.13,3.85)	-0.94 (-2.63,0.74)	-3.71 (-5.26,2.16)***
Follow guidelines extremely well	Reference		

* <0.05, ** <0.01, *** <0.001

## Discussion

This study assessed the mental health among cMSM in Los Angeles County from the HOPE study three months after the COVID-19 lockdown in March 19, 2020. This paper is among the first to study the mental health among cMSM in Southern California during the COVID pandemic. Taking advantage of the large HOPE cohort using social media as the intervention, we were able to reach out to participants at the beginning of the pandemic. We included several measures that have not previously been studied, e.g., COVID-19 pandemic related anxiety, belief in government efficiency, ever experienced food insecurity. We found that having a full-time job, being healthy, not using tobacco, having food security, using social media less than six hours per day, and having a higher belief score in government efficiency were significantly associated with a lower BDI depression score. We also found that being healthy, having food security, and having a higher belief score in government efficiency were significantly associated with lower GAD anxiety level and COVID-related anxiety. COVID-related anxiety level was higher among those who followed the government guidelines extremely well. This is consistent with findings that the massive media attention to this pandemic, inconsistency of the guidelines, and policy changes lead to higher levels of anxiety and uncertainty at the beginning of the pandemic (Bendau et al., 2020; Sigdel et al., 2020; Zakout et al., 2020).

The COVID-19 pandemic is the third recorded outbreak of coronavirus (Feehan & Apostolopoulos, 2021) after the SARS-CoV-1 in early 2002 and Middle East respiratory syndrome (MERS) in 2012. Given the unclear impact of COVID-19, it may be that here are long-term influences on health behavior and mental health (McBride et al., 2021). A study that evaluated the helpline calls related to COVID-19 from 19 countries found that the peak of call volumes were at six weeks after the initial outbreak, due to the fear of infection, loneliness, and health concerns (Brülhart et al., 2021). There have been many studies that have focused on the COVID-19 related mental health status locally and globally, but fewer studies have focused on MSM population. Due to the reduced access to HIV and sexually transmitted disease (STD) prevention services during the pandemic, an online cohort in the United States found that 25% of the participants discontinued their PrEP, HIV testing, and STD testing (Pampati et al., 2021) from October 2019 to July 2020. In western China, the incidence of anxiety using Anxiety Self-Rating Scale and depression using Center for Epidemiological Studies Depression (CES-D) Scale among MSM were 21.7% and 38.0% during the “post-pandemic period” (Pan et al., 2021). In our sample, we found that 30.7% had anxiety using GAD-7 and 23.0% had depression using BDI-21. A study in Brazil, which used the 5-item World Health Organization Well-Being Index, (WHO-5) found that low psychological well-being was associated with younger age, being in a polyamorous relationship, not complying with social isolations measures and unstable sexual relationships (Camargo et al., 2021). Another study conducted among Indonesian MSMs using The Kessler Psychological Distress (K10) and the Subjective Happiness Scale (SHS) found high level of psychological distress during the pandemic (69.1%) and 87.1% considered themselves not happy (Septarini et al., 2021).

COVID pandemic-related anxiety is an important psychosocial factor that is associated with behavior change and government guidelines. In general, racial and sexual minorities were more vulnerable during the lockdown in the United States because a lot of prevention programs and services were canceled to reduce physical contact (Patterson et al., 2020; Reyniers et al., 2020; Sanchez et al., 2020; Wiss et al., 2021). Although our study of cMSM was conducted in July, it indicated that mental health was significantly associated with financial status and employment status. Those who were more stable financially in the past month were less likely to report depression and anxiety. This suggests that intervention programs for MSM should focus on providing jobs and basic level of living expenses. We found that the majority of participants experienced food insecurity during the lockdown time. Food insecurity among MSM has been shown to be associated with lower rates of HIV testing (Nyirenda et al., 2018; Takada et al., 2020; Weinhardt et al., 2017; Wiss et al., 2021), higher new HIV infection (Amon Exavery et al., 2020), and potential violence in the community (Adhikari et al., 2020). It is important to improve the access to food secure care programs. HOPE aimed to promote the use of HIV home testing kits among participants. It is critical to provide this contactless free testing during the pandemic. In this paper, we did not find any significant differences on mental health outcomes between the intervention and control group. We also did not find significant differences on mental health outcomes between those who completed the study and those who are still in the cohort.

There were some limitations in this study. We did not survey all participants in the HOPE cohort. Instead, we used a convenience sample of the first 300 participants who responded quickly to our recruitment letter. We compared the baseline characteristics to check whether participants who participated in the study were different from the rest of participants in the HOPE cohort. With the exception of educational level being higher in this follow up survey, there were no other significant differences. This study was cross sectional and based on a snapshot of the COVID-19 pandemic in the middle of this long pandemic. It may not reflect the actual behavior change after the pandemic. Given the length of the pandemic and waves of policy changes, a longitudinal study is needed to assess the effects of COVID-19 on mental health among MSM. We have identified a need for a pandemic-focused and psychometric validated survey that can be used to evaluate the depression and anxiety that can be used to evaluate the mental health status after the outbreak of the pandemic. The 10-item questions used to measure the pandemic related anxiety were not from a psychometric validation questionnaire. Rather, the questions were adapted from an instrument that was validated in the context of H1N1 influenza in 2009–2010 (Wheaton et al., 2012) and the Zika virus pandemic during 2015–2016 (Blakey & Abramowitz, 2017).

## Conclusions

This study provides useful information for further development of mental health-related instruments targeted towards COVID-19. Hence, we will have better survey tools to prepare for the next pandemic. The findings from this study emphasize the importance of establishing trust between government and communities when issuing public health policies. The study findings can also be used to inform local communities and stakeholders in the ongoing and extensive efforts to address the urgent needs among disadvantaged populations. The factors that were associated with depression and anxiety may help local agencies develop programs and interventions that can target cMSM to help them recover from the pandemic more quickly. Consistent with the findings from other studies on the mental health among MSM, we suggest additional resources on the basic needs, including food security, as well as mental health support should be considered during the lockdown and later in the recovery efforts (Akré et al., 2021).

## Data Availability

The datasets generated during and/or analyzed during the current study are not publicly available due to confidentiality of the participants, but are available on reasonable request to Principal Investigator, Dr. Sean Young, email: syoung5@hs.uci.edu.

## Appendix

**Table S1:** Regression of categorical BDI-21 and GAD-7 using cutoff score for general population

		BDI-21		GAD-7
**Intervention**				
**No**		0.93 (0.48,1.79)		1.42 (0.8,2.52)
**Age**				
**One year increase**		0.97 (0.93,1.01)		0.97 (0.94,1)
**Education**				
**High School or less**		1.61 (0.63,4.15)		1.56 (0.65,3.73)
**Associate/Bachelor degree**		1.17 (0.51,2.7)		1.89 (0.9,3.96)
**Graduate and above**		Reference		
**Marital status**				
**Married/in a partnership**		0.87 (0.38,2.02)		0.69 (0.34,1.41)
**Single**		Reference		
**Employment**				
**Other**		1.87 (0.91,3.84)		1.02 (0.53,1.94)
**Part time**		1.54 (0.6,3.95)		0.84 (0.34,2.07)
**Full time**		Reference		
**General health**				
**Unhealthy**		3.09 (1.28,7.45)		2.65 (1.17,5.99)*
**Average**		1.19 (0.57,2.46)		1.27 (0.67,2.4)
**Healthy**		Reference		
**Use Tobacco**				
**Yes**		3.11 (1.57,6.16)**		1.84 (0.98,3.48)
**No**		Reference		
**Ever experienced food insecurity**				
**Yes**		2.97 (1.12,7.89)*		0.96 (0.91,1.01)***
**No**		Reference		
**Hours on social media/online communities**				
**4–8 h**		1.31 (0.64,2.67)		0.85 (0.45,1.6)
**More than 8 h**		3.03 (1.14,8.04)*		1.86 (0.74,4.69)
**<4 h**		Reference		
**Belief in government efficiency**				
**7-item score**		0.98 (0.93,1.04)		0.96 (0.91,1.01)
**Follow government guidelines**				
**Not at all**		0.88 (0.24,3.17)		1.04 (0.34,3.18)
**Moderately**		1.73 (0.75,4.03)		1.06 (0.48,2.35)
**Follow guidelines extremely well**		Reference		
